# The kinetic profile and clinical implication of SCC-Ag in squamous cervical cancer patients undergoing radical hysterectomy using the Simoa assay: a prospective observational study

**DOI:** 10.1186/s12885-020-6630-0

**Published:** 2020-02-21

**Authors:** Shuang Ye, Xiaohua Sun, Bin Kang, Fei Wu, Zhong Zheng, Libing Xiang, Mylène Lesénéchal, Fabienne Heskia, Ji Liang, Huijuan Yang

**Affiliations:** 1 Department of Gynecologic Oncology, Fudan University Shanghai Cancer Center, Shanghai, 200032 China; 2 Department of Oncology, Shanghai Medical College, Fudan University, Shanghai, China; 3 Fudan University Shanghai Cancer Center – Institute Merieux Laboratory, Cancer Institute, Fudan University Shanghai Cancer Center, Shanghai, China; 4 bioMerieux (Shanghai) Company Limited, Shanghai, 200032 China; 5 R&D Immunoassay Department, bioMerieux SA, Marcy l’Etoile, France; 6 Global Medical Affairs Department, bioMerieux SA, Marcy l’Etoile, France

**Keywords:** Squamous cervical cancer, Squamous cell carcinoma antigen, Simoa assay, Kinetic profile

## Abstract

**Background:**

To study the kinetic profile and clinicopathological implications of squamous cell carcinoma antigen (SCC-Ag) in cervical cancer patients who underwent surgery by a self-developed SCC-Ag single molecule assay (Simoa) prototype immunoassay.

**Methods:**

Participants were prospectively enrolled between 04/2016 and 06/2017. Consecutive serum samples were collected at five points: day 0 (the day before surgery), postoperative day 4, weeks 2–4, months 2–4 and months 5–7. In total, 92 patients and 352 samples were included. The kinetic change in SCC-Ag levels and their associations with clinicopathological characteristics were studied.

**Results:**

Simoa SCC-Ag was validated by comparison with the Architect assay. SCC-Ag levels measured by the Simoa assay were highly correlated with the Architect assay’s levels (Pearson’s correlation coefficient = 0.979, Passing-Bablok regression slope 0.894 (0.847 to 0.949), intercept − 0.009 (− 0.047 to 0.027)). The median values for each time-point detected by the Simoa assay were 2.49, 0.66, 0.61, 0.72, and 0.71 ng/mL, respectively. The SCC-Ag levels decreased dramatically after surgery and then stabilized and fluctuated to some extent within 6 months. Patients with certain risk factors had significantly higher SCC-Ag values than their negative counterparts before surgery and at earlier time points after surgery, while no difference existed at the end of observation. Furthermore, although patients with positive lymph nodes had sustained higher SCC-Ag levels compared to those with negative lymph nodes, similar kinetic patterns of SCC-Ag levels were observed after surgery. Patients who received postoperative treatment had significantly higher SCC-Ag values than those with surgery only at diagnosis, while no difference existed after treatment.

**Conclusions:**

The Simoa SCC-Ag prototype was established for clinical settings. The SCC-Ag levels were higher in patients with risk factors, whereas the kinetic trend of SCC-Ag might be mainly affected by postoperative adjuvant therapy. These data indicate that the SCC-Ag level might be a good predictor for the status of cervical cancer, including disease aggressiveness and treatment response.

## Background

Cervical cancer is the fourth most common female malignancy worldwide [1]. Each year, more than half a million women are diagnosed with cervical cancer, and the disease results in over 300,000 deaths [2]. Cervical squamous cell carcinoma (SCC), as the most common histologic subtype, accounts for approximately 70% of all cases [2, 3]. Squamous cell carcinoma antigen (SCC-Ag) is well known as the most useful marker for cervical squamous cell carcinoma [4, 5]. SCC-Ag was first isolated by conventional protein purification methods from a cervical squamous cell carcinoma [6]. Biochemical characterization of the original protein fraction (TA-4) showed that it comprised a group of proteins with a molecular weight of approximately 45 kDa. Currently, the most widely used SCC-Ag assay in clinical settings is proposed by Abbott on the Architect instrument (Abbott Laboratories, Abbott Park, IL, USA) [7, 8]. SCC-Ag assays are also available on other well-known platforms, such as the Elecsys® SCC assay used on the Roche Elecsys and cobase analyzer (Roche Diagnostics, China) [9].

The role of serum SCC-Ag in squamous cervical cancer has been extensively evaluated in previous works [4, 7, 8, 10–24], and several reviews and meta-analyses have been published in the literature [5, 25–27]. Most studies were of retrospective design and only detected SCC-Ag at one time-point. They could be roughly divided into two groups according to the SCC-Ag measurement time: first, the clinical relevance of pretreatment SCC-Ag, which is still debated [4, 5, 14, 15, 18, 20, 22–24], and second, the value of SCC-Ag in the monitoring of response to treatment and follow-up [7, 8, 17, 18, 20]. To date, few studies have investigated the dynamic change in serum SCC-Ag levels during treatment, from surgery to adjuvant therapies.

The single molecular array (Simoa) platform is a new ultrasensitive technology that allows for the measurement of very small amounts of proteins using a fully automated instrument to perform ELISA immunoassays [28, 29]. The fundamental theory of Simoa has been published by Chang and coworkers [30]. In cancer diagnostics, by utilizing Simoa, prostate-specific antigen (PSA) has a thousand-fold lower limit of quantification (< 0.01 pg/mL) than conventional ultrasensitive PSA assays, which allows for monitoring recurrence of prostate cancer after radical prostatectomy [31–33].

In the present study, we aim to develop and validate a Simoa SCC-Ag assay. Furthermore, we prospectively monitored serial SCC-Ag levels in patients during treatment and follow-up to determine the SCC-Ag profiles and clinicopathological implications.

## Methods

### Preparation of beads with capture and detection antibodies

Capture antibody (rabbit anti-human SerpinB3, 13,218-RP01) and detection antibody (rabbit anti-human SerpinB3, 13,218-T52) for the development of the Simoa SCC-Ag sandwich immunoassay were purchased from Sino Biological (Beijing, China). The preparation of beads with capture and detection antibodies followed the manufacturer’s protocol (Quanterix). The capture antibody concentration was adjusted to 0.2 mg/mL with Bead Conjugation Buffer (Quanterix), and then paramagnetic carboxylated microparticles (Quanterix) were activated with 0.3 mg/mL 1-ethyl-3-(3-dimethylaminopropyl) carbodiimide hydrochloride (EDC) (Thermo Fisher Scientific, Waltham, MA, USA). To start the biotinylation reaction, 3 μL of the biotin solution (2 mg of NHS-PEG4-Biotin dissolved in 383 μL of ddH_2_O) was added to 100 μL of the detection antibody solution (1.0 mg/mL). The concentration of the recovered antibody was adjusted to 0.2 mg/mL, and beads were stored at 4 °C.

### Simoa assay setup and reagent preparation

All Simoa measurements were performed on a fully automated Simoa HD-1 Analyzer (Quanterix). The beads coated with SCC-Ag capture antibody were diluted in Diluent Buffer to 500,000 per test. The SCC-Ag detection antibody was diluted in diluent buffer to a working concentration of 0.3 μg/mL. Streptavidin-β-galactosidase concentrate was diluted to a working concentration of 100 pmol/L. The assay configuration protocol was a two-step assay. In the first step, 25 μL of the microparticle solution, 20 μL of detection antibody and a 100-μL serum sample (two-fold manual dilution by sample diluent (Quanterix)) or calibrator were incubated for 35 min and 15 s (45 cadences) in a reaction cuvette (Quanterix), followed by several wash steps. In the second step, 100 μL of SBG was added and incubated for 5 min and 15 s (7 cadences), followed by several wash steps.

### Simoa assay validation procedure

During assay validation, the following basic assay parameters were addressed: calibration curve model, limit of quantification, sensitivity (lower limit of quantification, LLOQ), reproducibility (intra-assay, inter-assay), linearity, and calibrator stability.

To generate the calibration curve, recombinant human SCC-Ag (TP302683, Origene, USA) was serially diluted in the sample diluent, and the final concentrations of the 8 calibrators (calibrators A- H) in the assay were 0.049 to 50 ng/mL. The validation of the calibration curve model was performed by running six independent measurements, including the 8 calibrators in quadruplicates. The coefficient of variation (CV) was determined over all assay runs using the recalculated concentration values. Acceptance criteria were a recovery of initial values within 80–120% and a CV below 20% of all back-calculated calibrator samples. The reproducibility was assessed by two controls and four native human serum samples shared in the whole calibration curve. All samples were tested in quadruplicates over 6 runs on three different days (*n* = 24). The intra-assay and inter-assay CV% for each sample were lower than 20%. To determine the lower limit of quantification (LLoQ), 6 samples diluted in sample diluent to reach concentrations between zero and 0.1 ng/mL were measured over six independent measurements in quadruplicates, and the %CV was plotted as a function of SCC-Ag concentration to graphically determine the concentration when 20% CV was reached. Sixty-four replicates of the zero calibrator (sample diluent) were tested in several assay runs to assess the limit of blank (LoB). By ranking the concentrations of the 64 replicates of the zero calibrator in an increasing way, the LoB corresponding to the 95th percentile was the mean of the concentration between the 60th and the 61st. The limit of detection (LoD) concentration was 2.5 SD from the LoB. Linearity was evaluated by triplicate measurement within one run: three different human serum samples with high concentrations of SCC-Ag were mixed with a human serum sample with low concentrations of SCC-Ag at different proportions. Acceptance criteria were a recovery of the measured concentration within 80–120% by the nominal concentration. Calibrator stability was addressed by performing freeze-thaw and short-term stability tests. Aliquoted sets of calibrators were stored at 4 °C, − 20 °C, and room temperature for 1 week. In addition, one set of calibrators was frozen at − 20 °C and thawed at room temperature to obtain calibrators with one additional freeze-thaw cycle and then stored at − 20 °C for 1 week. To determine the short-term temperature stability, the prepared four sets of calibrators were tested in parallel 1 week later. Acceptance criteria were a recovery of initial values within 80 and 120%.

### Patients and treatment

After obtaining approval from the Institutional Review Board at Fudan University Shanghai Cancer Center (1703170–5), we prospectively enrolled the patients with SCC scheduled for radical hysterectomy surgery in Professor Huijuan Yang’s team (Department of Gynecologic Oncology, Fudan University Shanghai Cancer Center, FUSCC) from April 2016 to June 2017. The inclusion criteria were as follows: 1) preoperative confirmation of squamous histology; 2) International Federation of Gynecology and Obstetrics (FIGO 2009) stage IB1-IIA2; 3) no preoperative treatment including chemotherapy and radiation; and 4) the ability to have strict follow-up visits in our center. Patients with any skin disorder or past cancer history were excluded. Written informed consent was acquired from all the participants included in the study. Each patient was supposed to have five consecutive blood samples collected at different points: day 0 (the day before surgery), day 4 (postoperative day 4) and follow-up periods (weeks 2–4, months 2–4 and months 5–7). In our clinical practice, the stage of patients’ cervical cancer was determined by two gynecologic oncologists by pelvic examination, according to the FIGO 2009 guideline [34]. Radical hysterectomy was performed according to Querle & Morrow (type C). Regarding postoperative adjuvant treatment, pelvic external beam radiotherapy (EBRT) and concurrent platinum-based chemotherapy were given to patients with intermediate- and high-risk factors according to the Sedlis criteria [35]. Intermediate-risk factors include lymphovascular space invasion (LVSI), deep stromal invasion and tumor size, while high-risk factors refer to positive margin, lymph node metastasis and positive parametria. Extended-field EBRT was delivered to those with positive common iliac lymph node or para-aortic lymph node. Systematic chemotherapy (carboplatin + paclitaxel) was administered to patients with more than two lymph node metastases after radiation. Routinely, the patients with cervical cancer in our institution are required to have regular follow-up visits after operation: every 3 months for the first 2 years, every 6 months in the next 3 years, and annually thereafter.

### Statistical analysis

Regression analysis was used to determine the correlation between the Simoa SCC-Ag assay and the Architect SCC-Ag assay (R package mcr, version 1.2.1) [36]. The Kruskal-Wallis test was used to test whether SCC-Ag measurements at different time points were different between patient groups defined by categorical clinical factors (lymph node metastasis, LVSI), stromal invasion and FIGO stage). Associations between categorical clinical factors were analyzed using the chi-squared test. All statistical tests were performed using the R package compareGroups (version 3.4.0) [37].

Relationships between important clinical factors and the overall SCC-Ag profile were studied with the generalized additive modeling (GAM) method to accommodate the nonlinear trend of SCCA-Ag over time. GAM allows for approximating nonlinear processes with smoothing functions as follows:

$$ h(y)={\beta}_0+f(t)+{\beta}_1{x}_1+{\beta}_2{x}_2+\dots +{\beta}_p{x}_p+\varepsilon $$

where function *f* represents a nonlinear function of time *t* and can be of any form. *x*_*i*_ and *β*_*i*_, *i = 1, 2, …, p*, represent other clinicopathological covariates and corresponding coefficients. In this study, nonparametric splines were used to approximate the nonlinear SCC-Ag profile over time. Interactions between clinicopathological factors and the smooth function are allowed to evaluate the association between clinicopathological factors (lymph node metastasis and adjuvant treatment) and the SCCA profile. GAM was performed in the R statistical environment with the package mgcv (version 1.8.17) [38].

## Results

### Assay development and validation

A bead-based immunoassay was developed for the measurement of human SCC-Ag using Simoa technology (Quanterix). The immunoassay development process included the evaluation of a suitable antibody pair and the optimization of assay conditions, such as the assay buffer composition, incubation times, and applied reagent concentrations.

Various antigens and antibodies were tested for the selection of a suitable calibrator protein and antibody pair with high affinity for SCC-Ag in sandwich immunoassays (results not shown). The best assay performance was achieved when 13,218-RP01 (Sino Biological) and 13,218-T52 (Sino Biological) were used as the capture antibody and detection antibody, respectively. Assay conditions were optimized (results not shown), and evaluation was based on the calibration curve and human SCC-Ag serum samples. The best performances were obtained in a two-step assay.

**Fig. 1:**
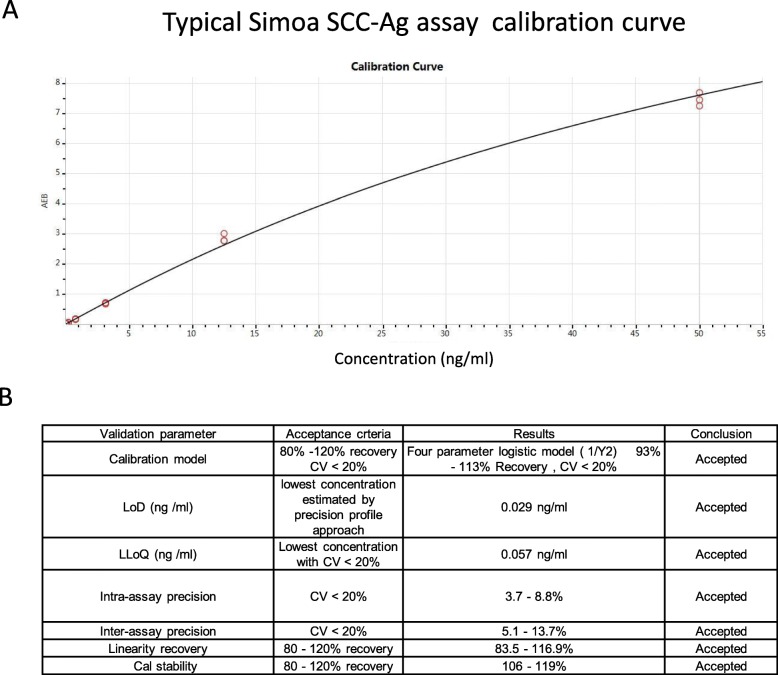
Simoa SCC-Ag assay calibration curve and validation. **a** Typical Simoa SCC-Ag assay calibration curve. Recombinant human SCC-Ag was serially diluted, and the calibrator range was 0.049 to 50 ng/mL with a recovery of all back-calculated concentrations between 80 and 120%. The fitting model for the calibration curve was a weighted four-parameter logistics (1/Y^2^). AEB: Average enzyme per bead (measured signal). **b** Validation results and acceptance criteria

During assay validation, the following basic assay parameters were addressed: calibration curve model, detection capability (LoB, LoD, and LoQ), reproducibility (intra-assay and inter-assay), linearity, and calibrator stability. The best fitting model for the calibration curve was the 1/Y^2^ weighted four-parameter logistics model. The recovery of all back-calculated concentrations of the individual calibrator points was between 93 and 113%. A typical Simoa SCC-Ag immunoassay calibration curve is given in Fig. 1a.

The Simoa SCC-Ag assay’s LoD and LoQ were calculated, and they achieved 0.029 and 0.057 ng/ml, respectively, according to the method of precision profile (Fig. 1b). The Simoa SCC-Ag assay’s LoD was approximately 4-fold lower than 0.1 ng/ml, the Architect SCC-Ag assay’s sensitivity. Inter-assay reproducibility for 6 samples resulted in CVs between 3.7 and 8.8%, and intra-assay repeatability for these samples resulted in CVs between 5.1 and 13.7% (Fig. 1b). The linearity of human serum samples with high and low concentrations of SCC-Ag showed a recovery between 83.5 and 116.9% over the working range (Fig. 1b). Under the optional standard curve, the dynamic range of the Simoa SCC-Ag assay was up to 0.029–100 ng/mL. Calibrator stability tests showed that they would be stable at − 20 °C with a recovery between 106 and 119%.

The Simoa SCC-Ag assay fulfilled acceptance criteria for all addressed validation parameters considered in the commonly used guidelines from the Clinical and Laboratory Standards Institute (CLSI). The method validation demonstrated that the required reproducibility and reliability for the measurement of complex matrices, such as human serum, were met by the Simoa SCC-Ag assay.

### Patient characteristics

During the study period, we enrolled 92 patients undergoing radical hysterectomy after receiving informed consent. For different reasons, some participants missed one or more points’ blood collection (please refer to Fig. 3 patient numbers for specific details). Therefore, a total of 352 blood samples from the 92 enrolled patients were measured and analyzed.

Table 1 presents the clinicopathological characteristics of the participants. The median age was 51 years old (range 32–71). The median level of presurgery SCC-Ag detected by the Simoa assay was 2.49 ng/mL (range 0.31–71.75). The FIGO stage (2009) of the patients is listed as follows: IB1 30.4%, IB2 10.9%, IIA1 32.6%, and IIA2 26.1%. Approximately 37% (34/92) of the patients presented with bulky tumors (> 4 cm). Deep stromal invasion, positive LVSI and lymph node metastasis accounted for 77.2, 58.7, and 35.9%, respectively. For the entire cohort, 45 (48.9%) patients received postoperative adjuvant treatment.

**Table 1:** Clinicopathological features of the participants (*n* = 92)

Median age(range), years	51(32–71)
Pre-surgery SCC-Ag (ng/ml)	2.49(0.31–71.75)
FIGO stage	
IB1 (%)	28(30.4%)
IB2 (%)	10(10.9%)
IIA1 (%)	30(32.6%)
IIA2 (%)	24(26.1%)
Tumor size > 4 cm (%)	33(35.9%)
Stromal invasion > 1/2 depth (%)	71(77.2%)
LVSI positive (%)	54(58.7%)
Lymph node metastasis (%)	33(35.9%)
Adjuvant treatment (%)	45(48.9%)

*Abbreviations*: *SCC-Ag* Squamous cell carcinoma antigen, *FIGO* International Federation of Gynecology and Obstetrics, *LVSI* Lymph-vascular space invasion

### Method comparison between the Simoa and Architect SCC-Ag assays

**Fig. 2:**
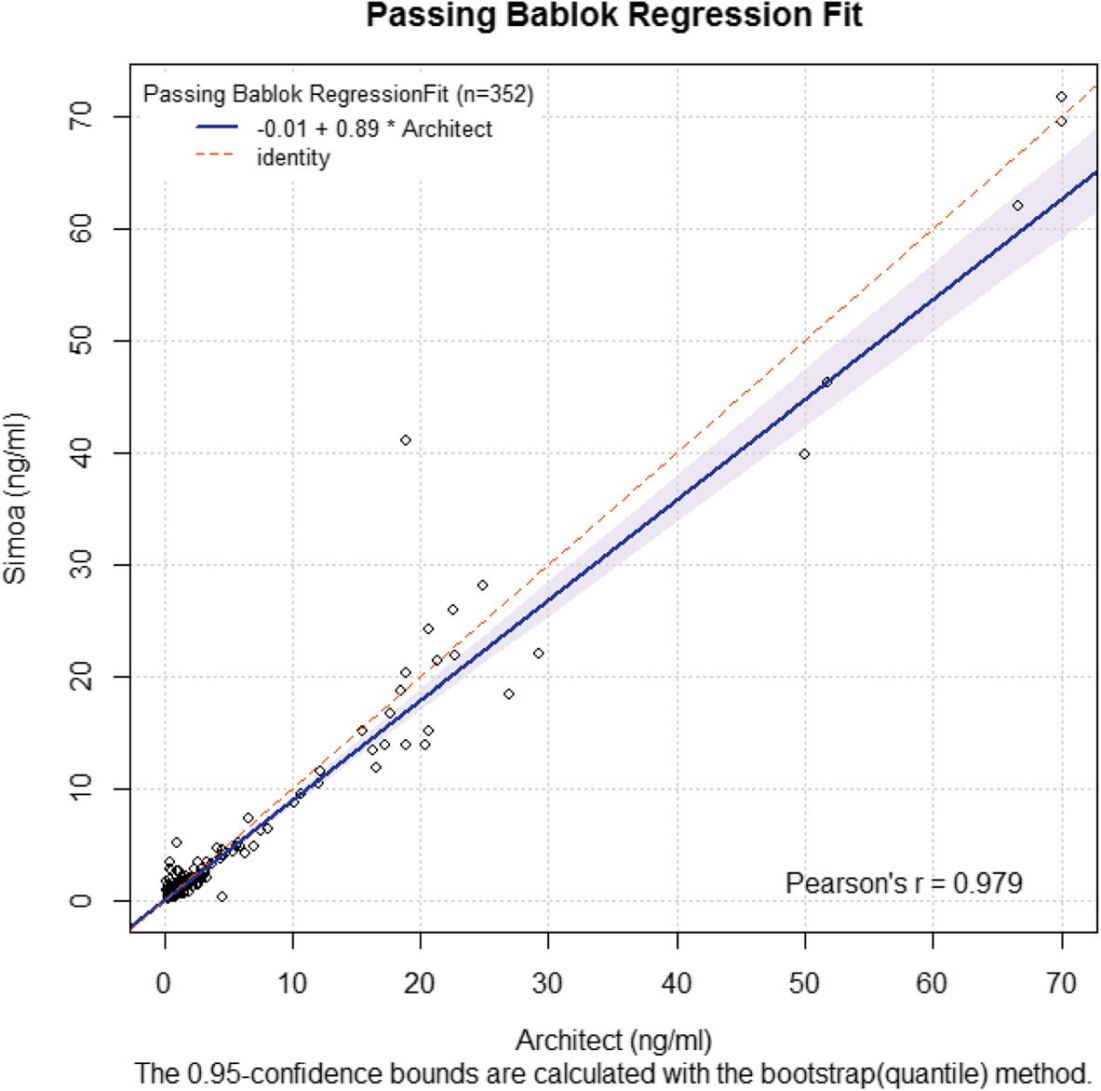
Passing–Bablok regression analysis of the SCC-Ag concentration of 352 samples obtained with the Architect and the Simoa SCC-Ag assay. Scatter diagram with regression line (blue line) and 95% confidence bands (light blue) for the regression line. Pearson correlation coefficient (R) of 0.979 (*p* < 0.001). Passing–Bablok regression line equation: y = 0.89x − 0.01 (intercept 95% confidence interval (CI): − 0.05 to 0.03; slope 95% CI: 0.85 to 0.95

Among the 352 samples tested on the Simoa platform, all were also tested with the Architect SCC-Ag assay. A comparison between the two methods was conducted to estimate the difference between the Simoa and Architect assays. SCC-Ag levels measured by the Simoa assay were highly correlated with the Architect assay’s levels (Pearson’s correlation coefficient = 0.979) (Fig. 2). The slope and intercept for the Passing-Bablok regression were 0.894 (0.847 to 0.949) and − 0.009 (− 0.047 to 0.027), respectively. The minimum values of SCC-Ag in the Architect and Simoa platform were 0.17 and 0.16 ng/mL, respectively (data not shown), and no sample had an SCC-Ag value lower than the sensitivity of both assays.

### Kinetic SCC-Ag data after surgery

**Fig. 3:**
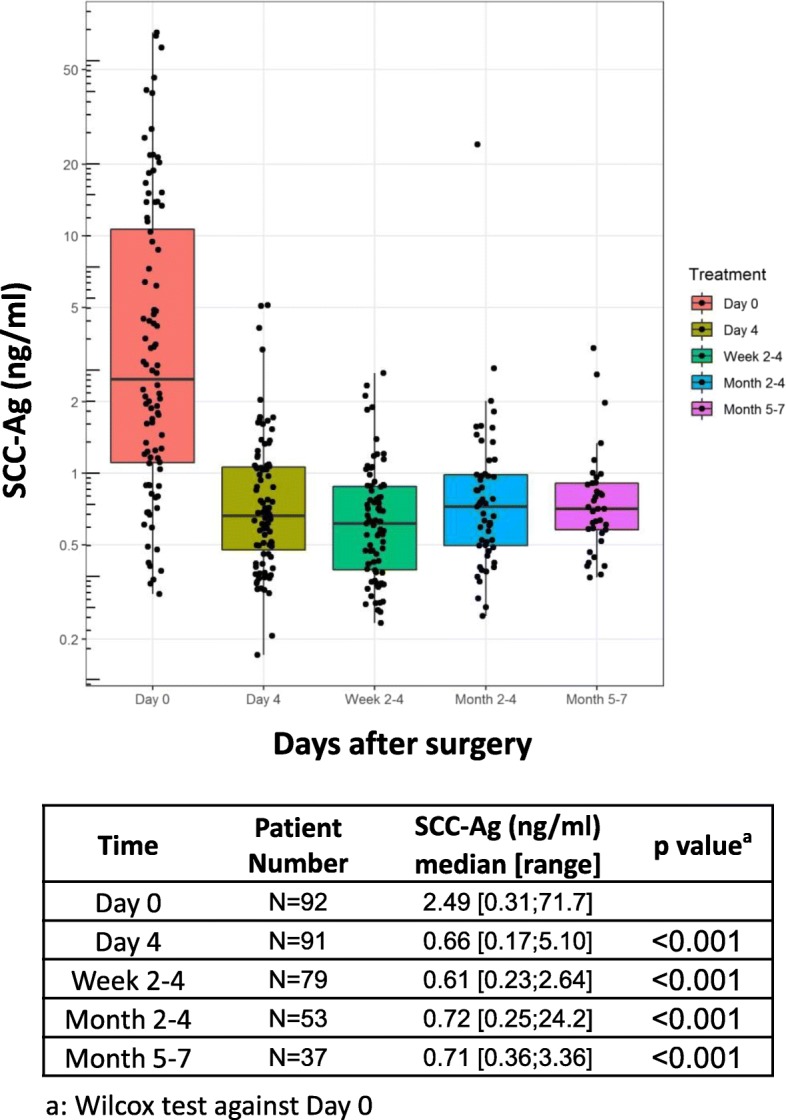
Simoa SCC-Ag median values and range at each time point (day 0, day 4, weeks 2–4, months 2–4, months 5–7)

The median SCC-Ag values and ranges for each time-point (day 0, day 4, weeks 2–4, months 2–4, months 5–7) are summarized in Fig. 3. The median SCC-Ag values for each time-point using Simoa were 2.49 ng/mL, 0.66 ng/mL, 0.61 ng/mL, 0.72 ng/mL, and 0.71 ng/mL. As shown in Fig. 3, the SCC-Ag values decreased dramatically after surgery and then stabilized.

**Fig. 4:**
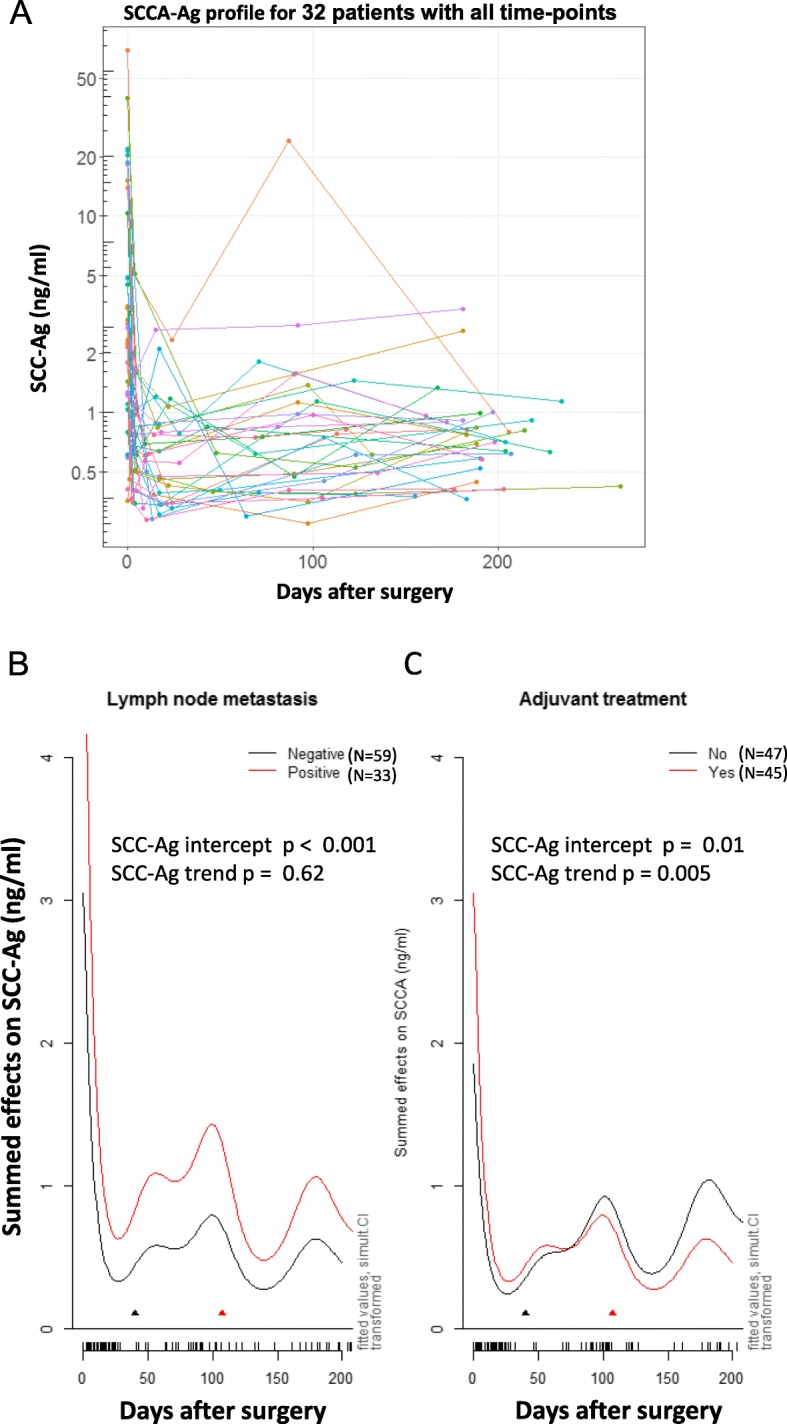
SCC-Ag profile analysis. **a** Profiles of SCC-Ag values for the 32 patients with all five time points. Each curve represents the SCC-Ag profile for one patient. **b** GAM analysis of the effect of lymph node metastasis on the SCC-Ag profile. The black arrow points to the start of adjuvant treatment, while the red arrow indicates the end of adjuvant treatment. SCC-Ag intercept p < 0.001, SCC-Ag trend *p* = 0.62. **c** GAM analysis of the effect of postoperative adjuvant treatment on the SCC-Ag profile. The black arrow indicates the start of adjuvant treatment, and the red arrow indicates the end of adjuvant treatment. SCC-Ag intercept *p* = 0.01, SCC-Ag trend *p* = 0.005

Among 92 patients, 32 patients succeeded in collecting samples at all time points. Figure 4a depicts the profiles of the SCC-Ag values for the 32 patients. All patients showed a sharp decrease in the SCC-Ag level after radical surgery, and then the SCC-Ag level began to change relatively slowly. In some patients, the SCC-Ag level began to increase within 1 month after surgery. In other patients, the SCC-Ag level reverted and increases during 1–3 months. In some patients, the SCC-Ag level remained decreased or stabilized until 3 months.

### Association between the SCC-Ag level and the Clinicopathological features

We evaluated the association between the SCC-Ag values and clinicopathological characteristics. As shown in Table 2, the presurgery SCC-Ag level was related to the FIGO stage, stromal invasion, and lymph node metastasis with statistical significance, but not to LVSI (*P* = 0.074). After surgery, we noted that patients with positive LVSI and lymph node had higher SCC-Ag levels at the time points of day 4 and weeks 2–4 than those without. The different levels of SCC-Ag between these groups did not reach statistical significance at months 2–4 and months 5–7. These results indicate that the presurgery SCC-Ag level might reflect the tumor burden and that the postoperative SCC-Ag level mainly indicates tumor metastasis through lymph drainage. Interestingly, the SCC-Ag levels reached the same level between the low-risk group, intermediate-group and high-risk group after completion of treatment.

**Table 2:** Correlation of clinical information with Simoa SCC-Ag values at different time points

	LVSI			Lymph node metastasis			FIGO stage			Stromal invasion		
	Negative	Positive	*p* value^*a*^	Negative	Positive	*p* value^*a*^	IB	IIA	*p* value^*a*^	<= 1/2	> 1/2	*p* value^*a*^
	*N* = 38	*N* = 54		*N* = 59	*N* = 33		N = 38	N = 54		*N* = 21	*N* = 71	
SCC-Ag (ng/ml), median[range]												
Pre-surgery	1.98 [0.34;71.7]	4.22 [0.31;69.6]	0.074	1.76 [0.31;71.7]	8.72 [0.61;69.6]	**< 0.001**	1.40 [0.31;69.6]	3.88 [0.36;71.7]	**0.003**	1.10 [0.34;13.9]	3.49 [0.31;71.7]	**< 0.001**
Day 4 post-surgery	0.55 [0.17;1.72]	0.72 [0.21;5.10]	**0.048**	0.57 [0.17;5.10]	0.93 [0.33;5.06]	**< 0.001**	0.65 [0.17;5.06]	0.68 [0.32;5.10]	0.353	0.57 [0.17;1.19]	0.68 [0.21;5.10]	**0.041**
Week 2–4 post-surgery	0.47 [0.27;2.64]	0.69 [0.23;2.34]	**0.013**	0.52 [0.23;2.64]	0.78 [0.29;2.34]	**0.003**	0.57 [0.23;2.34]	0.63 [0.27;2.64]	0.371	0.69 [0.27;2.12]	0.61 [0.23;2.64]	0.858
Month 2–4 post-surgery	0.59 [0.25;2.77]	0.85 [0.30;24.2]	**0.019**	0.67 [0.25;2.77]	0.85 [0.30;24.2]	0.123	0.68 [0.25;24.2]	0.75 [0.35;2.77]	0.336	0.68 [0.30;1.45]	0.75 [0.25;24.2]	0.539
Month 5–7 post-surgery	0.54 [0.36;3.36]	0.77 [0.52;2.60]	0.117	0.66 [0.36;3.36]	0.72 [0.52;2.60]	0.273	0.71 [0.37;1.00]	0.71 [0.36;3.36]	0.477	0.58 [0.37;1.14]	0.71 [0.36;3.36]	0.415

*Abbreviations*: *SCC-Ag* Squamous cell carcinoma antigen, *FIGO* International Federation of Gynecology and Obstetrics, *LVSI* Lymph-vascular space invasion

^a^Kruskal-Wallis test

### SCC-Ag kinetic trends according to lymph node metastasis and postoperative treatment

The above results reveal that patients with positive and negative lymph nodes had significantly different SCC-Ag levels within 1 month after surgery but not after 2–4 months. In our cohort, all patients with lymph node metastasis received postoperative adjuvant therapy. Thus, the SCC-Ag kinetic trend was further evaluated based on lymph node metastasis and postoperative treatment using the generalized additive modeling (GAM) technique. After controlling for age, tumor size, and adjuvant treatment, significantly elevated SCC-Ag levels were associated with positive lymph node (*P* < 0.001) (Fig. 4b). Moreover, a trend analysis showed that the kinetic trends of SCC-Ag over time were similar for patients with or without lymph node metastasis (*P* = 0.62). Moreover, higher SCC-Ag levels were associated with postoperative adjuvant treatment after controlling for age, tumor size, and lymph node status. However, the kinetic trends of SCC-Ag levels were significantly changed (*P* = 0.005) between postoperative adjuvant-treated and nontreated patients (Fig. 4c). The trend-altering effect of postoperative treatment was further demonstrated by ANOVA. Although patients with postoperative adjuvant treatment had significantly higher SCC-Ag levels than patients without at the beginning of the treatment, the difference between the two groups disappeared after completion of adjuvant treatment (two-way ANOVA *p* = 0.56). All these data indicated that the SCC-Ag levels detected by the Simoa assay are a good predictor of disease aggressiveness and the treatment response of cervical cancers.

## Discussion

In recent years, the Simoa platform has been proven to be an ideal tool for clinical implementation due to its simple and fully automated manipulation and ultra-sensitive detection limit [28, 29]. In the current study, a new prototype of sensitive SCC-Ag immunoassay was developed using Simoa technology. This assay fulfilled the acceptance criteria for all addressed analytical parameters and demonstrated improved sensitivity compared to that of the Architect assay, the most commonly used method. Molecular cloning has demonstrated that SCC-Ag is produced by two almost identical genes named SCCA1 (SerpinB3) and SCCA2 (SerpinB4) [39]. In spite of controversy, most studies agreed that SCCA1 is more relevant for SCC diagnosis, and the Architect assay only detects SCCA1 but not SCCA2 [40–42]. In our study, our Simoa prototype also detected SCCA1 antigen.

Researchers have investigated the clinical significance of consecutively monitoring the level of serum SCC-Ag in cervical cancer patients during radiation/chemoradiation therapy [11, 17, 21]. Hashimoto et al. evaluated the value of SCC-Ag as a predicator of chemotherapy response in patients with metastatic cervical cancer and concluded that a response to chemotherapy was possible for patients in whom SCC-Ag levels declined between the second and third cycles of chemotherapy [17]. Markovina et al. found that persistently elevated serum SCC-Ag during definitive chemoradiation therapy was an independent predictor of positive posttherapy FDG-PET/CT, recurrence and death [11]. However, until now, few studies have addressed the dynamic change in SCC-Ag value in patients receiving radical surgery. To our knowledge, we are the first to describe the kinetic change in SCC-Ag levels before and after radical hysterectomy surgery within a six-month duration. We found that the SCC-Ag values stabilized after the dramatic drop in the first few immediately after surgery. In the dot plot graph (Fig. 3), the lowest SCC-Ag median value was observed at the time point of weeks 2–4, although significance was not achieved. After that nadir point, some patients exhibited fluctuations, while others reached a plateau. It deserves further investigation whether different patterns correlated with treatment and survival outcome.

In the second part of our work, we examined the relationship between the SCC-Ag values and clinicopathologic features. Not surprisingly, the pretreatment SCC-Ag level was related to tumor aggressiveness as indicated by advanced stage, deep stromal invasion and lymph node metastasis, which was consistent with previous works [5, 19]. Most published studies focused on the clinical value of one time-point of SCC-Ag and both the pretreatment level [10–12, 14, 15, 20, 22, 23], and posttreatment level [7, 8, 13–16, 18, 24]. Here, we monitored SCC-Ag values in a longitudinal way to try to understand the possible clinical meaning of the SCC-Ag levels. Our new finding was that patients with intermediate- and high-risk factors had higher SCC-Ag levels postoperatively, while the difference became insignificant 6 months after surgery. As patients with risk factors received adjuvant treatment after surgery, we further evaluated the impact of postoperative treatment on the SCC-Ag pattern. Patients with positive lymph nodes before surgery showed sustained elevated levels of SCC-Ag compared to those negative counterparts, while the two groups had similar overall SCC-Ag tendencies. In contrast, although patients who received adjuvant therapy had raised baseline SCC-Ag levels, no difference existed at the completion of treatment. In short, we postulated that the absolute levels of SCC-Ag might be determined by the disease severity, while the dynamic change was possibly influenced by postoperative adjuvant treatment.

Given the short follow-up time, we did not evaluate the prognostic value of the SCC-Ag level in cervical cancer patients. A recent retrospective study with a large sample size from our institution demonstrated that a preoperative serum SCC-Ag level > 2.75 ng/mL is an independent prognostic factor for progression-free survival in cervical squamous cell carcinoma patients with high-risk factors [23]. In addition, a recent study investigated the association between posttreatment SCC-Ag levels and survival in patients treated with concurrent chemoradiation [24]. Patients with posttreatment SCC-Ag ≥ 1.8 ng/mL had significantly poor survival [24].

The study has several limitations. First, not all patients completed the five-point blood collection for various reasons. Second, we prospectively enrolled 92 participants, which was not a large sample size. Finally, given the short-term follow-up, no survival outcome was analyzed in the current work, which deserves further assessment.

## Conclusion

The Simoa SCC-Ag assay exhibited competitive analytical performances when compared with the Architect SCC-Ag assay. The profile of SCC-Ag after radical surgery was illustrated for the first time. Both pre- and postoperative SCC-Ag values are good predictors for tumor aggressiveness with different clinical applications. In addition, postoperative SCC-Ag is an effective response factor for adjuvant treatments following radical surgery.

## Data Availability

The dataset supporting the conclusions of this article is available upon request. Please contact Prof. Huijuan Yang (huijuanyang@hotmail.com).
